# Oxidative Unzipping and Transformation of High Aspect Ratio Boron Nitride Nanotubes into “White Graphene Oxide” Platelets

**DOI:** 10.1038/srep29498

**Published:** 2016-07-08

**Authors:** Pranjal Nautiyal, Archana Loganathan, Richa Agrawal, Benjamin Boesl, Chunlei Wang, Arvind Agarwal

**Affiliations:** 1 Plasma Forming Laboratory, Department of Mechanical and Materials Engineering Florida International University, Miami, FL 33174, USA; 2 Energy Materials Laboratory, Department of Mechanical and Materials Engineering Florida International University, Miami, FL 33174, USA

## Abstract

Morphological and chemical transformations in boron nitride nanotubes under high temperature atmospheric conditions is probed in this study. We report atmospheric oxygen induced cleavage of boron nitride nanotubes at temperatures exceeding 750 °C for the first time. Unzipping is then followed by coalescence of these densely clustered multiple uncurled ribbons to form stacks of 2D sheets. FTIR and EDS analysis suggest these 2D platelets to be Boron Nitride Oxide platelets, with analogous structure to Graphene Oxide, and therefore we term them as “White Graphene Oxide” (WGO). However, not all BNNTs deteriorate even at temperatures as high as 1000 °C. This leads to the formation of a hybrid nanomaterial system comprising of 1D BN nanotubes and 2D BN oxide platelets, potentially having advanced high temperature sensing, radiation shielding, mechanical strengthening, electron emission and thermal management applications due to synergistic improvement of multi-plane transport and mechanical properties. This is the first report on transformation of BNNT bundles to a continuous array of White Graphene Oxide nanoplatelet stacks.

The brilliant mechanical, thermal and electrical properties of Carbon Nanotubes (CNT) propelled the scientific community towards exploration of this light-weight material for diverse applications over the past two decades^1^. One major shortcoming of CNTs is their poor high temperature stability; they start oxidizing around 400 °C^2^,^3^, limiting their applications to lower temperatures. Boron Nitride Nanotube (BNNT), a structural analogue of CNT, displays a unique combination of exceptional strength and flexibility with low density, thermal conductivity coupled with electrical insulation, piezoelectric behavior, radiation shielding effect and high temperature stability^2^,^3^,^4^,^5^. BNNTs are resistant to oxidation at temperatures as high as 800–900 °C (twice the temperature for CNT)^2^,^3^. This makes BNNT greatly desirable for numerous applications, *viz.* light weight aircraft and space vehicle bodies which are usually subjected to tremendous aerodynamic heating, thermal management of high density power electronics systems, high temperature piezoelectric sensors and high temperature field emitters^4^,^5^,^6^,^7^,^8^,^9^,^10^,^11^,^12^,^13^.

Despite the fanfare around superior oxidation resistance, there is very little understanding of BNNT behavior at elevated temperatures. Chen and co-workers^2^ found that the oxidation performance of BNNT depends on their nanostructure. Coarse nanotubes, such as bamboo-like and cone-shaped BNNTs exhibited broken walls, whereas thin nanotubes retained their multiwalled structure on exposure to high temperatures. In addition, thin nanotubes were found to withstand higher temperatures (upto 900 °C) without oxidizing. Golberg *et al*.^3^ fabricated BNNT ropes comprising of multiwalled nanotubes and investigated high temperature behavior of these ropes. First signs of oxide formation were noticed only after 800 °C. While these studies report that BNNT reacts to form Boron Trioxide (B_2_O_3_) around 750–900 °C^2^,^3^, the oxidative transformation mechanism of BNNTs remains elusive. Being a 1-D nanomaterial, BNNT is anisotropic with strong directionality in its properties. This makes the understanding of morphological and phase transformations associated with oxidation critical for high temperature applications.

In this article, we unravel the oxidative transformations of high aspect ratio (~30,000–70,000) BNNT bundles. We report atmospheric O_2_ induced cleavage and unzipping of BNNTs, followed by coalescence of the unwrapped nanotubes to form 2D platelet stacks at temperatures exceeding 750 °C. These plates have analogous structure to Graphene Oxide, and therefore we term them as “White Graphene Oxide” (WGO)^14^,^15^. However, not all BNNTs deteriorate even at temperatures as high as 1000 °C. This leads to the formation of a hybrid nanomaterial system comprising of 1D BN nanotubes and 2D BN oxide platelets at elevated temperatures. This is the first report on high temperature oxidative unzipping of BNNTs to form white graphene oxide.

## Results

**Figure 1:**
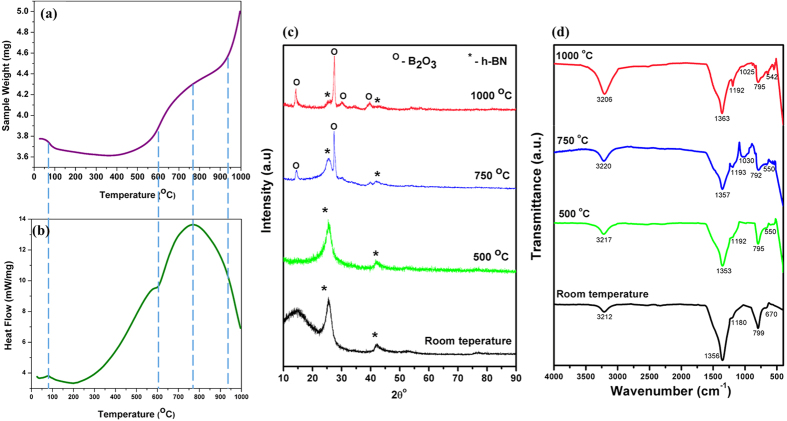
**(a)** Weight change of BNNT and **(b)** heat required to increase the temperature of BNNT sample during TGA-DSC, as a function of temperature. **(c)** Post thermal treatment phase characterization by X-Ray Diffraction, and **(d)** Fourier Transform Infrared Spectrum (FTIR) of BNNT sample subjected to TGA-DSC for varying temperatures, ranging from room temperature to 1000 °C.

Thermal analysis of nanotube clusters was performed by simultaneous Thermogravimetric Analysis (TGA) and Differential Scanning Calorimetry (DSC) tests up to 1000 °C. Major peaks/transition points are marked in TGA (Fig. 1a) and DSC (Fig. 1b) curves. There is an abrupt drop in weight initially, until ~80 °C, after which the weight loss becomes more gradual. This transition is characterized by a sharp peak in the DSC curve in the beginning, which is associated with the loss of moisture in the sample. Vaporization is an endothermic process, which explains the peak in DSC curve. There is a dominant peak at ~750 °C, signifying major phase transition. A continuous weight gain trend is seen in the TGA curve in this regime, which can be related to the initiation of BNNT oxidation. Generalized Gradient Approximation (GGA) calculations performed by Zhang *et al*. for (3, 0) BNNT predicted lower oxidation energy barrier of 0.138 eV/atom, which corresponds to ~790 °C^16^. The possibility of the oxidation reaction is subsequently probed by XRD, FTIR and EDS analysis.

X-Ray Diffraction (XRD) analysis of BNNT before and after TGA-DSC thermal treatment up to peak temperatures of 500, 750 and 1000 °C was performed to closely monitor the phase evolution and the progression of the oxidation reaction (Fig. 1c). Presence of the characteristic h-BN peaks for all the temperatures indicates survival of h-BN phase up to 1000 °C. However, the peak intensity begins decreasing from 750 °C, indicating oxidative transformation of the BN phase. There is no B_2_O_3_ phase observed at 500 °C. A Peak corresponding to B_2_O_3_ was detected for the first time in the BNNT sample heated up to 750 °C. This is in agreement with the DSC curve (Fig. 1b), where a peak value was noticed at ~750 °C and can be attributed to the initiation of h-BN transformation to B_2_O_3_. The intensity of the B_2_O_3_ signal is enhanced at 1000 °C, where other B_2_O_3_ peaks are also visible (Fig. 1c), suggesting greater concentration of boron trioxide due to the accelerated rate of oxidation at high temperatures. This is attested by TGA curve (Fig. 1a), where there is a rapid increase in weight gain after 900 °C (characterized by a steep rise in TGA slope). The weight gain can be explained by the higher density (~2.46 g/cm^3^) of B_2_O_3_, as compared to h-BN (~2.1 g/cm^3^). In fact, the BNNT fibrils used in this study are ultra-light, with an unusually low density of ~0.5 mg/cm^3^ 17. This explains more than 31% weight gain at the end of TGA (1000 °C) with respect to as-received BNNTs noticed in Fig. 1a. Disintegration of lightweight nanotubes at high temperatures and formation of comparatively denser non-tubular BN based phase and B_2_O_3_ leads to the overall weight gain.

FTIR plots in Fig. 1d reveal characteristic B-N stretching (~1353–1363 cm^−1^) and bending modes (~792–799 cm^−1^)^18^ for the entire temperature range investigated, showcasing the impressive oxidation resistance of these high aspect ratio nanotubes. It is interesting to note that the peaks corresponding to the B-O band (1192 cm^−1^) and O-B-O bond (550 cm^−1^)^18^ appear in BNNT treated at 500 °C. However, there is no B_2_O_3_ phase detected in XRD for this condition (Fig. 1c). Such behavior can be attributed to chemisorption of O_2_ on B atoms of nanotubes at 500 °C, without any free oxide formation. At 750 and 1000 °C, an additional peak corresponding to B-O stretching (1025–1030 cm^−1^) is detected. At these temperatures, B_2_O_3_ formation has been confirmed from XRD. Therefore, BN-O_2_ interactions are not just limited to surface adsorption; rather, an oxidation reaction takes place to form boron trioxide. In addition, peaks corresponding to B-OH (3206–3220 cm^−1^)^18^ were also detected. This peak is very weak at room temperature (in as-received material), and could be due to moisture. However, at elevated temperatures, there is a marked increase in peak intensity. This suggests B-OH bond formation. This is corroborated by the gradual increase in weight in the TGA curve (Fig. 1a), suggesting progressive attachment of -OH functional group to h-BN as the temperature increases before B_2_O_3_ formation initiates.

**Figure 2:**
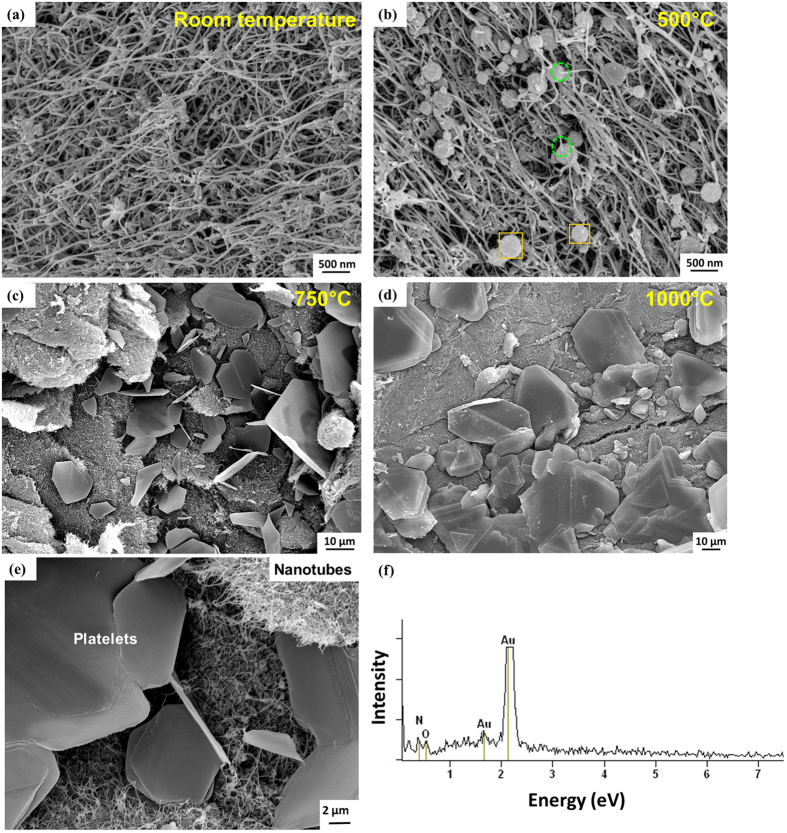
SEM images of BNNT sample at different TGA-DSC maximum temperatures showing: **(a)** pure nanotube cluster at room temperature, **(b)** growth of nodules in nanotube network at 500 °C, **(c)** formation of thin stacks of 2-D plates while retaining nanotube clusters at 750 °C, and **(d)** the formation of dense and thick 2-D stacks of white graphene oxide, with further reduction in the amount of nanotube clusters. **(e)** High magnification image showing the co-existing nanotube and platelet network. **(f)** EDS spectrum of platelets revealed O (Note: Au peaks are due to sputter coating for SEM examination of insulator BNNTs. B and H cannot be detected by EDS due to their low atomic number, and therefore is not marked here).

Transformations taking place in shape, morphology, and elemental composition of BNNT as a function of TGA-DSC temperature were examined by scanning electron microscopy (SEM) and Energy Dispersive Spectroscopy (EDS). Figure 2a shows the BNNTs at room temperature, consisting of a densely entangled network of fine and long nanotubes. Very small spherical particles can also be seen. These are possibly B and BN nanoparticles, which are reported in BNNTs synthesized by HTP technique^19^. After heating up to 500 °C, these spherical nodules were observed to grow in size as large as 400 nm (Fig. 2b). Some of the nodules were attached to the tubes (encircled in green), while others grow in isolation from BNNTs. EDS examination of nodules revealed the presence of O in these locations. The presence of B-O bonds detected in FTIR (Fig. 1d) suggests that the growth of these nodules is related to the chemisorption of O_2_, and subsequent surface reaction on the nanoparticles.

The heating of BNNTs to 750 °C resulted in the formation of thin 2D plates, while most of the nanotubes remain untransformed (Fig. 2c). This shows that some of the BNNTs unwrap at elevated temperature to form planar ribbons, which coalesce to form 2D plates. At 1000 °C, the thickness of the stacked plates further increases (Fig. 2d), suggesting accelerated oxidative transformation of nanotubes. This is attested by the TGA curve (Fig. 1a), where there is a steep increase in weight gain after 900 °C. There were no spherical nodules in 750 and 1000 °C treated samples, indicating complete oxidation of the nanoparticles to form B_2_O_3_. XRD and FTIR results (Fig. 1c,d) suggest the survival of h-BN phase even after the BNNT transform to the plate structure. Oxygen was detected on EDS examination of these plates (Fig. 2f). Based on FTIR and EDS results, it is clear that these 2D plates have B-O bonds, in addition to h-BN bonds. Moreover, B-OH bonds were also detected in FTIR (Fig. 1d). Therefore, these 2D plates, characterized by B-O and B-OH linkages attached to an array of B-N sheets, have a structure analogous to Graphene Oxide, which also consists of different oxygen based functional groups attached to parent graphite structure^14^. Therefore, it can be concluded that BNNTs undergo high temperature oxidative unzipping to form Boron Nitride Oxide^15^, a structural analogue of Graphene Oxide (GO). Hence, we term it as ‘White Graphene Oxide’ (WGO).

## Discussion

**Figure 3:**
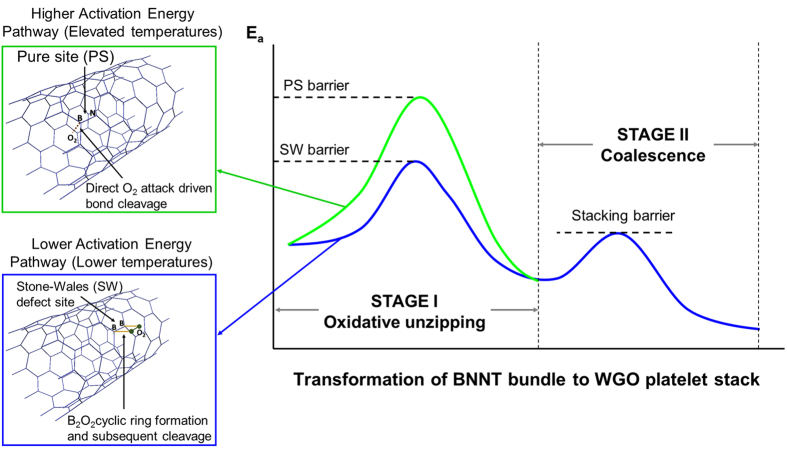
Schematic diagram showing two-stage BNNT to WGO transformation at elevated temperatures. Activation barrier for oxidative unwrapping of BNNTs via Stone Wales defect site and pure site is compared.

The oxidation resistance of these ultra-long (up to ~200 μm) and fine nanotubes (3–6 nm) is very high. While morphological transformation of BNNTs starts at 750 °C, it is seen that even after heating up to 1000 °C, a significant portion of nanotubes remains intact (Fig. 2e). This suggests that the activation barrier for complete nanotube oxidation is certainly higher than 1000 °C, or else the tubular structures should have completely disintegrated. Local density approximation (LDA) calculations by Zhang *et al*. for (3, 0) BNNT predicts the upper limit of this barrier to be as high as ~1500 °C^16^, hinting to the possibility of the presence of defect sites in the nanotubes which preferentially oxidize at lower temperatures. BNNTs are known to exhibit Stone-Wales (SW) defects^20^, which are characterized by the presence of B-B and N-N homonuclear bonds. In fact, the weak 670 and 1180 cm^−1^ peaks in IR spectrum of the BNNTs at room temperature are suggestive of the presence of B-B and N-N bonds, respectively (Fig. 1d)^21^,^22^. These defect sites are reactive due to local strain and bond frustration^20^; therefore the corresponding peaks vanish at higher temperatures. O_2_ is reported to undergo dissociative chemisorption near these SW sites. While chemisorption in a pure site is an endothermic process (reported bond energy ~1.39–1.6 eV), chemisorption of molecular oxygen near Stone Wales defect sites is an exothermic process (reported bond energy ~−1.76 to −2.38 eV)^23^. Therefore, the selective oxidation and transformation of nanotubes is possibly related to defects. Chemisorption of molecular O_2_ at SW site typically takes place at B atom, forming a cyclic B_2_O_2_ ring structure^23^, as shown in Fig. 3. At higher temperatures, thermal vibration energy is expected to be quite high, causing B-B bonds of a B_2_O_2_ ring to cleave resulting in the formation of a strong B-O chemical bond, as also observed in FTIR results. The activation barrier for breaking such a strained 4-member ring is expected to be low (schematically shown in Fig. 3). Due to the strain induction, this bond cleavage extends along the entire BNNT^24^, resulting in *unzipping* of the tube to form a planar nanoribbon^24^,^25^,^26^,^27^,^28^,^29^,^30^,^31^,^32^,^33^. Contrary to this, pure sites have tendency for physisorption instead of chemisorption due to the mismatch of atomic orbitals of O_2_ with that of BNNT^23^. Chemisorption, therefore, is likely to take place at a pure site only under drastic conditions, such as high temperatures. Complete disintegration of tubular structures will take place only after the barrier for B-O chemical bond formation/B-N bond dissociation is exceeded, which appears to be higher than 1000 °C for the nanotubes studied here (Fig. 3).

**Figure 4:**
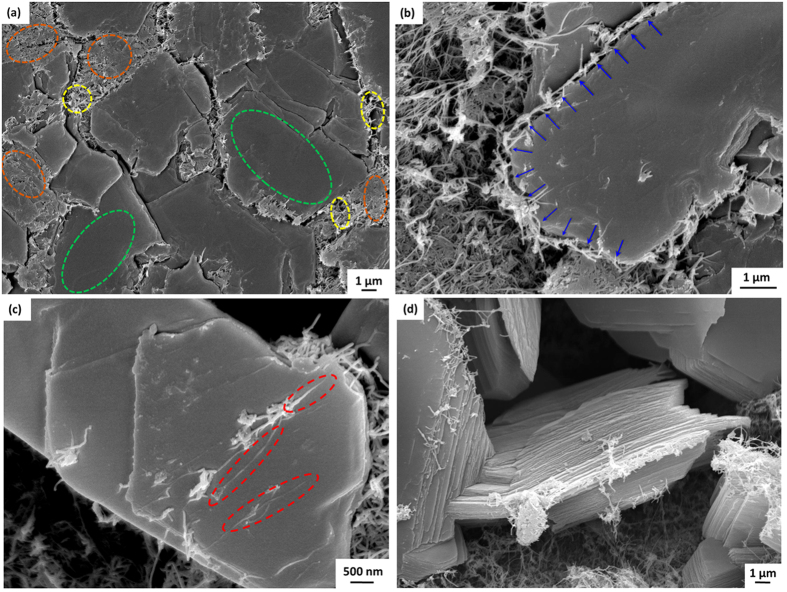
SEM images showing progression of oxidative transformation of BNNT: **(a)** unreacted, partially uncurled and completely transformed regions marked yellow, orange and green respectively, **(b)** sideways growth of 2D WGO platelet by unzipping and coalescence of BNNTs, **(c)** uncurled nanoribbon forming a new peel over previous layer in WGO stack, and **(d)** fully formed thick stack of WGO layers, with some dangling nanotubes visible along the sideways.

The mechanism of formation of white graphene oxide (WGO) platelets can be explained in two major steps: (i) high temperature oxidative unzipping of BNNTs, and (ii) coalescence of the unfolded nanoribbons to form a continuous network of 2D plates (schematically shown in Fig. 3). This can be seen in the SEM image of the BNNT sample subjected to TGA-DSC heating up to 750 °C (Fig. 4a). The regions encircled in yellow are unreacted BNNTs, retaining their fine tubular structure. Regions encircled in orange consist of nanotubes unwrapped to form planar ribbons, which are in the process of coalescing together to form 2D plates. Regions marked in green show fully formed, integrated and continuous 2D plates of WGO.

**Figure 5:**
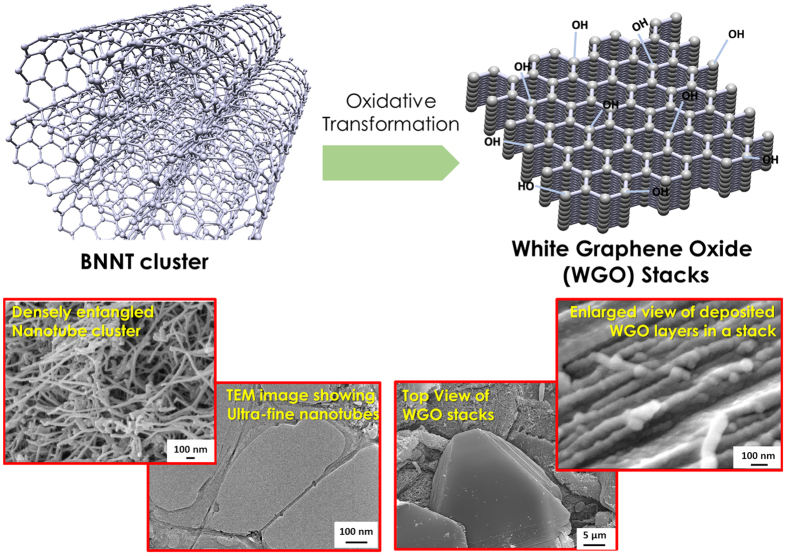
Schematic representation of transformation of nanotube clusters to 2D stacks of white graphene oxide, with accompanying representative SEM/TEM images.

The WGO platelets grow with time and temperature. These 2D plates grow by sideways expansion as well as stacking of new planar layers over the existing ones. The mechanism of sideway growth of these 2D plates is elucidated in Fig. 4b, where the transformed plates are surrounded by unreacted nanotubes. These BNNTs will unzip and merge with the parent plate, resulting in the growth of continuous array of planar WGO layers (shown with blue arrows). Figure 4(c) shows the unzipped and partially cleaved BNNTs in the process of forming a new peel over the previous layer. The tubular/semi-tubular structures encircled in red represent the positions where nanotubes unwrap and start forming an ultra-thin 2D layer at the top, forming stacks of WGO. Figures 4a–c correspond to a BNNT sample after 750 °C TGA-DSC heating. A fully formed, thick stack of WGO layers at 1000 °C is shown in Fig. 4d. This is demonstrative of multi-layered architecture consisting of 2D plates formed by high temperature BNNT unzipping and coalescence. The transformation is schematically shown in Fig. 5, accompanied with SEM images. High resolution image of White Graphene Oxide plates stacked to form a 2D architecture is shown.

**Figure 6:**
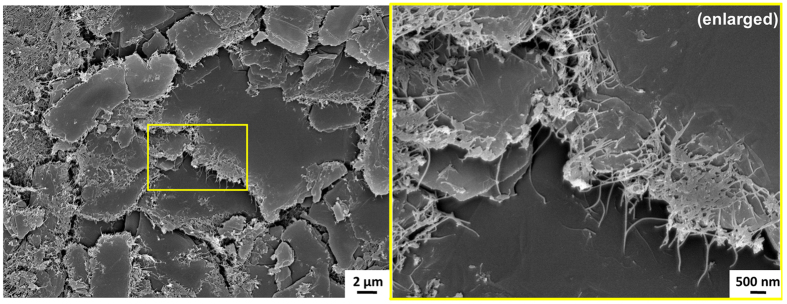
SEM image of BNNT sample after thermal treatment at 750 °C showing 3 D architecture, comprising of multiple 2D plates interconnected by nanotubes. Zoomed view (right) clearly shows vertical nanotubes acting as pillars interconnecting the layers of 2D platelets.

Some of the untransformed nanotubes can also be seen dangling from the stack edges in Fig. 4d. Based on these observations, the possibility of a hybrid architecture consisting of parallel WGO layers with vertical nanotube pillars can be envisioned by careful engineering of the material system. The feasibility of such a unique nanostructure anatomy is shown by means of the SEM images at 750 °C in Fig. 6. Figure 6 clearly contrasts unzipped platelets from the untransformed nanotubes.

## Conclusion

In summary, oxidative unzipping of BNNTs takes place at temperatures exceeding 750 °C. Multiple uncurled nanotubes in a dense cluster coalesce together to form stacks of 2D white graphene oxide platelets. Even for temperatures as high as 1000 °C, significant proportion of nanotubes remain untransformed, confirming high oxidation resistance of BNNTs. The presence of nanotubes as well as platelets leads to a hybrid (1D + 2D) nanomaterial system. Mechanical, thermal and functional performance of such a system would be enhanced due to improved out-of-plane properties, and has direct implications in high temperature sensing, BNNT based heat conduction systems, Al-BNNT composite by casting route, mechanical load transfer, radiation shielding and spintronic applications of BNNT. This is the first report on the formation of stacks of white graphene oxide platelets by BNNT unzipping at elevated temperatures.

## Methods

Long boron nitride nanotube clusters obtained from BNNT, LLC were used in this study. These nanotubes were fabricated by High Temperature-Pressure (HTP) method^17^. Simultaneous Thermogravimetric Analysis (TGA) – Differential Scanning Calorimetry (DSC) tests were performed on BNNT sample in SDT Q600 for three different values of maximum temperature: 500, 750 and 1000 °C. Samples were tested in air at the scan rate of 5 °C/min. Phase evolution was studied by X-Ray Diffraction of thermally treated samples for all the three peak temperature conditions using Siemens D-5000 X-ray diffractometer (Munich, Germany). Chemical transformations were examined by Fourier Transform Infrared Spectroscopy, using JASCO FT-IR 4100.

Morphological transformations were studied by scanning electron microscopy (SEM) of the thermally treated BNNT samples, using JEOL JSM-6330F field emission SEM (Tokyo, Japan), corresponding to each TGA-DSC test. Elemental analysis of key morphological features was performed by Energy Dispersive Spectroscopy (EDS).

## Additional Information

**How to cite this article**: Nautiyal, P. *et al*. Oxidative Unzipping and Transformation of High Aspect Ratio Boron Nitride Nanotubes into “White Graphene Oxide” Platelets. *Sci. Rep.* **6**, 29498; doi: 10.1038/srep29498 (2016).
